# Fused Deposition Modeling of ABS-Barium Titanate Composites: A Simple Route towards Tailored Dielectric Devices

**DOI:** 10.3390/polym10060666

**Published:** 2018-06-14

**Authors:** Bilal Khatri, Karl Lappe, Mathis Habedank, Tobias Mueller, Christof Megnin, Thomas Hanemann

**Affiliations:** 1 Laboratory for Materials Processing, University of Freiburg, 79110 Freiburg, Germany; lappe@posteo.de (K.L.); mhabedank@gmx.de (M.H.); thomas.hanemann@kit.edu (T.H.); 2 Institute of Automation and Applied Informatics, Karlsruhe Institute of Technology, 76344 Eggenstein-Leopoldshafen, Germany; tobias.mueller2@kit.edu; 3 Institute of Applied Materials, Karlsruhe Institute of Technology, 76344 Eggenstein-Leopoldshafen, Germany; christof.megnin@kit.edu

**Keywords:** rapid prototyping, polymer-ceramic composites, fused deposition modeling, material characterization, mechanical characterization, dielectric characterization

## Abstract

A process for the development, characterization and correlation of composite materials for 3D printing is presented, alongside the processing of a polymer-ceramic functional composite using fused deposition modeling (FDM). The composite was developed using acrylonitrile butadiene styrene (ABS) as the matrix material filled with barium titanate (BT) micro-powder up to 35 vol % (74.2 wt %). The ABS-BT composites exhibited a shear thinning behavior with increasing ceramic content. The composite was 3D printed into structural and functional test samples using FDM by adapting and optimizing the print parameters. Structural characterization revealed increasingly brittle behavior at higher filler ratios, with the ultimate tensile strength falling from 25.5 MPa for pure ABS to 13.7 MPa for the ABS-35 vol % BT composite. Four-point flexural tests showed a similar decrease in flexural strength with increasing ceramic content. Functional characterization revealed an increase in the relative permittivity at 200 kHz from 3.08 for pure ABS to 11.5 for the composite with 35 vol % BT. These results were correlated with the Maxwell-Garnett and Jayasundere-Smith effective medium models. The process described in this work can be used for other 3D printing processes and provides a framework for the rapid prototyping of functional composites into functional parts with reliable properties. The ABS-BT composite shows promise as a functional dielectric material, with potential applications as capacitors and light-weight passive antennas.

## 1. Introduction

Under the umbrella of additive rapid prototyping (ARP) or 3D printing, several technologies, including stereolithography (SL), selective laser sintering (SLS) and polyjet modeling (PJM), enable the bottom-up, accurate, free-form fabrication of parts and structures [1] traditionally produced using subtractive methods such as milling, drilling and cutting. The most well-known of these is fused deposition modeling (FDM) or fused filament fabrication (FFF), emerging as a highly versatile and promising ARP technology due to its relatively low material and running costs. First developed by Stratasys Inc. (Eden Prairie, MN, USA) in the late 1980s, FDM has traditionally used heat-assisted structuring of polymer filaments into 3D parts in a layer-by-layer manner and has found applications in areas as diverse as casting [2], tissue engineering [3,4,5,6], optics [7,8], microfluidics [9], antenna design [10,11], food production [12,13] and electronics [14,15].

The versatility of ARP lies in the possibility of using composite materials; i.e., traditionally 3D-printable polymeric ‘matrix’ materials combined with one or more ‘filler’ materials that exhibit a functional property for a specific application. Several recent investigations have looked at ceramic suspensions for SL [16,17,18,19,20,21,22,23], alongside glass- [24] and magnetite-based [25] composites.

The use of composite materials for FDM has increased significantly in the past two decades [26,27,28]. Numerous studies have looked at the material characteristics of the parts produced by FDM [29,30,31], as well as the preparation and characterization of composite materials for FDM, including polymer-carbon [32,33], polymer-metal [34,35,36,37,38,39,40] and polymer-ceramic [41,42,43,44] composites. It is clear that ARP has come into its own and is rapidly evolving into a family of reliable manufacturing processes for the production of unique and specialized parts, both for prototyping and for low-volume production [45,46].

**Figure 1:**
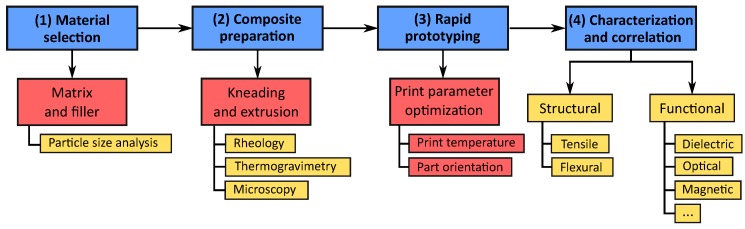
A schematic describing the various phases in the preparation, structuring and characterization of polymer-based composites using ARP. Blue: The four phases in the process, starting with (1) the selection of appropriate constituent materials, followed by (2) the preparation of the composite, through (3) 3D printing to (4) the characterization of the printed samples and correlation with existing data or a theoretical model. Red: The process steps used in this work for the ABS-BT composites. Yellow: The corresponding characterization for each of the process steps including investigations into the material, structural and functional properties of the composite.

In this work, a comprehensive four-phase process is described for the development and characterization of functional composites for ARP as illustrated in Figure 1. This process can be adapted to work with any ARP technology by the use of appropriate development and characterization methods for each phase.

The process starts with the selection of the matrix material and a functional filler. The matrix material could be a thermoplastic for FDM or an acrylic monomer for SL, whereas the filler could be a metal, ceramic, semiconductor or any other functional material. The materials have to be stabilized and homogenized before being characterized for their material properties, such as rheology, thermogravimetry, calorimetry or microscopy. The material properties can then be adjusted to work within the boundaries of the ARP process through additives and agents. The third phase is the production of test samples, followed by their structural and functional characterization. The results obtained can then be correlated back with existing theoretical models for composite materials. This four-phase process can be adapted to work with other ARP technologies by employing the appropriate processing and characterization methods.

This work uses the ARP process for a polymer-ceramic composite comprised of acrylonitrile butadiene styrene (ABS) as the polymer matrix loaded with barium titanate (BT) micro-powder for FDM. It follows up on an earlier investigation into the ARP of a polymer-metal composite [39] using a similar process. The ABS-BT composites were prepared in filler ratios of up to 50 vol % by kneading and characterized for their rheological properties. The composite feedstock with up to 35 vol % BT was extruded into filaments with a defined diameter to ensure compatibility with a commercial FDM 3D printer. Thermogravimetric analysis was performed on the feedstock and filaments to investigate the composite’s filler content at each process step. Building upon a preliminary examination [47] of the ABS-BT composite, the print temperature and orientation of the test samples were optimized for the composites during the third process phase. Structural test samples were printed and characterized for their tensile and flexural properties. Functional characterization investigated the relationship of the filler content on the relative permittivity of the composites and was correlated with the Maxwell-Garnett [48] and Jayasundere-Smith [49] effective medium models.

The process, starting from composite preparation, through filament production, printing and characterization, shows repeatable and reliable results at each stage and is only limited by the rheology of the composites and the mechanical limitations of the 3D printer.

## 2. Material Preparation

### 2.1. Feedstock Preparation

The polymer-ceramic composites were prepared using injection molding-grade acrylonitrile butadiene styrene (ABS) pellets (Terluran^®^GP-22, Styrolution, Frankfurt, Germany) and 3 μm BT microparticles (Sigma-Aldrich, now a part of Merck KGaA, Darmstadt, Germany).

**Figure 2:**
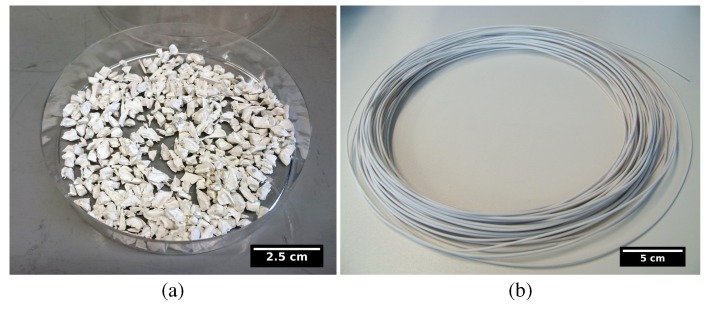
(**a**) A photo of the ABS-BT feedstock after kneading. (**b**) An ABS-BT composite filament sample.

The ABS pellets were kneaded with the BT particles in ratios of 10, 20, 30, 35, 40, 45 and 50 vol % with up to a maximum of 1.1 wt % steric acid as a surfactant, forming the composite feedstock. This step was carried out in the Brabender W50-EHT kneader (Brabender GmbH, Duisburg, Germany) for 60 min at 30 rpm. Figure 2a shows a photo of a feedstock sample.

#### Filament Extrusion

**Figure 3:**
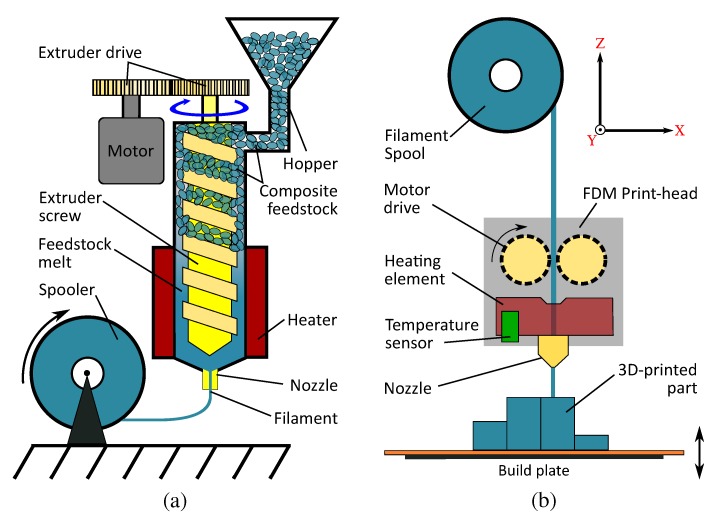
(**a**) A schematic depiction of the filament extrusion process. The kneaded composite material chunks are forced down a heated barrel using a screw, extruded through a nozzle of a defined diameter and spooled. (**b**) The FDM printing process: A motor drive forces the filament into the temperature-controlled print-head, mounted on an XY stage. After being forced through a nozzle, the material is deposited on to a build plate, which moves down to allow for the structuring of the next layer.

The ABS-BT kneaded feedstock were dried in an oven for 24 h at 130 °C in an ambient atmosphere to minimize moisture. They were then extruded into filaments using the Noztek Pro single barrel extruder (Noztek, Shoreham, UK) between 185 and 210 ${}^{\circ }$C. Figure 3a illustrates the filament extrusion process. Filament diameters in the range of 1.75 ± 0.10 mm were realized by adjusting the spooling speed for each composite, as shown in Figure 2b. Composites with filler ratios over 35 vol % were challenging to extrude due to their rheology.

### 2.2. 3D Printing

The composite filaments were structured into three different kinds of test specimens using the MakerBot Replicator 2X 3D printer (MakerBot Industries, New York, NY, USA). Pure ABS test samples were also printed for comparison. Figure 3b shows the FDM printing process. The print-head, mounted on an XY-stage, heats the incoming filament to beyond its glass transition temperature. The material is forced through a nozzle of a defined diameter (0.3 mm) and is deposited onto a build platform. After the completion of a layer, the build platform recedes by a defined amount, and the next layer can then be deposited.

## 3. Sample Design and Characterization

### 3.1. Structural Characterization

Tensile and flexural samples were characterized in the Zwick/Roell Z010 universal testing machine (Zwick/Roell, Ulm, Germany). Five samples were characterized for each point on each of the tests. Microscopy was performed on the specimen cross-sections to investigate the particle distribution and the presence of air pockets.

#### 3.1.1. Tensile

**Figure 4:**
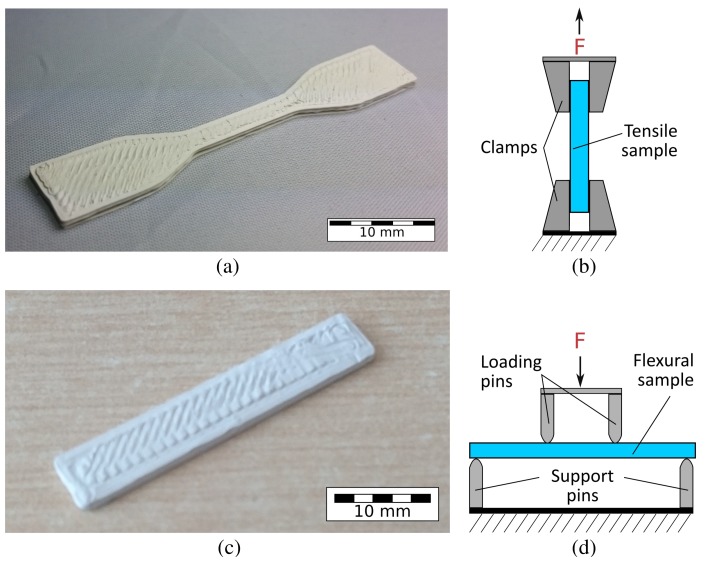
(**a**) A 3D-printed tensile specimen based on the ASTM-D638 (Type IV) standard. (**b**) A schematic of the tensile testing method. The test specimen is clamped on both ends and pulled at a constant velocity, while the force and displacement are measured simultaneously. (**c**) A 3D-printed flexural test specimen based on the ASTM-D7264M standard. (**d**) A schematic depiction of the flexural testing setup. The loading pins, having been spaced one-third of the support span, press down on the specimen till mechanical failure, while the applied force and deflection are measured.

Tensile specimens were based on the ASTM-D638 (Type IV) standard, with dimensions (L) 57 mm × (B, center) 3.5 mm × (H) 1.5 mm. Figure 4a,b shows a tensile specimen and a schematic of the tensile testing process, respectively. The samples were tested until failure to investigate the effect of the ceramic filler on the ultimate tensile strength (UTS) and the Young’s modulus of the composites.

#### 3.1.2. Flexural

Four-point flexural tests were carried out in accordance with the ASTM D7264M standard, with dimensions (L) 30 mm × (B) 5.5 mm × (H) 1.5 mm for composites up to 30 vol % BT. The load-span was set to one-third of the support span. Figure 4c,d shows a test specimen and the testing schematic, respectively.

The flexural strength was calculated using the relation,

(1) $$\sigma_{f}\;=\;\frac{FL}{bh^{2}},$$

where $\sigma_{f}$ is the maximum flexural stress experienced by the material, *F* is the applied force, *L* is the support span length, *b* is the specimen width and *h* is the thickness.

The flexural secant modulus of elasticity was calculated at one-fifth of the maximum deflection for each specimen using the equation,

(2) $$E_{f}^{secant}\;=\;\frac{0.17L^{3}m}{bh^{3}},$$

where $E_{f}^{secant}$ is the flexural secant modulus of elasticity, *L* is the support span length, *m* is the slope of the force-deflection curve at one-fifth of the maximum deflection, *b* is the specimen width and *h* is the thickness.

### 3.2. Functional Characterization

**Figure 5:**
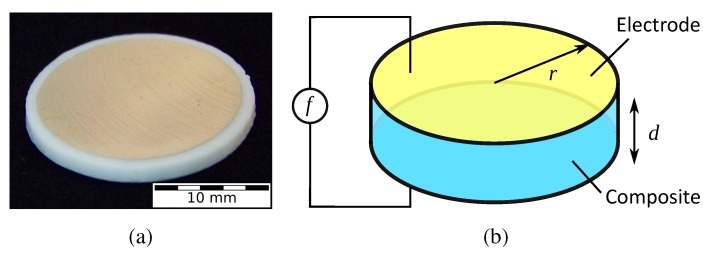
(**a**) A test specimen for dielectric analysis, with sputtered gold electrode on top. (**b**) Schematic of the dielectric characterization method, using the parallel plate capacitor model.

To analyze the dielectric properties of the composites, disc-shaped 3D-printed specimens with a radius of 11 mm and thickness of 2 mm were prepared. The top and bottom surfaces of the specimens were first polished in the Buehler Phoenix 4000 polisher (Buehler GmbH, Braunschweig, Germany), followed by sputtering of 100 nm-thick gold electrodes on both sides. Figure 5a shows a photo of a dielectric test specimen.

The dielectric specimens were approximated to parallel-plate capacitors, as shown in Figure 5b, with edge effects ignored due to their high surface-to-volume ratio. Capacitances were measured for five samples of each composite over a frequency sweep from 250 Hz–200 kHz using the GW Instek LCR-821 (Instek, New Taipei City, Taiwan) combined with a spring-loaded parallel plate testing setup. These capacitances were then calculated back to the relative permittivity using the relation,

(3) $$\epsilon_{r}\;=\;\frac{Cd}{\epsilon_{o}A},$$

where $\epsilon_{r}$ is the relative permittivity of the composite material, *C* is the measured capacitance, $\epsilon_{o}$ is the permittivity of free space, *d* is the specimen thickness and *A* is the cross-section area.

The measured permittivities were correlated with the Maxwell-Garnett (MG) effective medium model for spherical ceramic inclusions in a polymer matrix [48,50] using,

(4) $$\epsilon_{c}\;=\;\epsilon_{m}\cdot \frac{2\epsilon_{m}+\epsilon_{f}+2v_{f}\left(\epsilon_{f}-\epsilon_{m}\right)}{2\epsilon_{m}+\epsilon_{f}-v_{f}\left(\epsilon_{m}-\epsilon_{f}\right)},$$

and with the Jayasundere-Smith (JS) effective medium model, first proposed for binary piezoelectric composites [49,51] and later used for epoxy-BT composites [52], given by,

(5) $$\epsilon_{c}\;=\;\frac{\epsilon_{m}v_{m}+\;\epsilon_{f}v_{f}\left(\frac{3\epsilon_{m}}{\epsilon_{f}\;+\;2\epsilon_{m}}\right)\left(1+3v_{f}\frac{\epsilon_{f}\;-\;\epsilon_{m}}{\epsilon_{f}\;-\;2\epsilon_{m}}\right)}{\epsilon_{m}+v_{f}\left(\frac{3\epsilon_{m}}{\epsilon_{f}\;+\;2\epsilon_{m}}\right)\left(1+3v_{f}\frac{\epsilon_{f}\;-\;\epsilon_{m}}{\epsilon_{f}\;-\;2\epsilon_{m}}\right)},$$

where $\epsilon_{c}$, $\epsilon_{f}$ and $\epsilon_{m}$ are the relative permittivities of the composite, the matrix and the filler, respectively, and $v_{f}$ and $v_{m}$ are the volume fractions of the filler particles and the matrix, respectively.

The MG and JS models were correlated using the datasheet permittivity of ABS GP-22 ($\epsilon_{m}$ = 2.9) and $\epsilon_{f}$ = 2300 for BT, considering its dependency on temperature and grain size [53,54,55].

An additional correlation was carried out with both models by calculating the measured volume fraction of voids in samples of each composite variant through the measured masses and densities of the samples using,

(6) $$v_{f(air)}\;=\;\frac{m_{f(air)}}{m_{f(air)}+\left(1-m_{f(air)}\right)\frac{\rho_{air}}{\rho_{comp}}},$$

where $v_{f(air)}$ is the volume fraction of voids, $m_{f(air)}$ is the measured mass fraction of air, $\rho_{air}$ is the density of air (1.225 × 10${}^{-3}$ g/cm${}^{3}$ and $\rho_{comp}$ is the average density of the sample considering $\rho_{ABS}$ = 1.04 g/cm${}^{3}$, $\rho_{BT}$ = 6.02 g/cm${}^{3}$.

The void volumes were added to the matrix part of the MG and JS models ($\epsilon_{air}$ = 1.0006) to investigate the effect of the inherent void formation of FDM on the part’s functional performance.

## 4. Material Characterization

### 4.1. Particle Size Distribution

**Figure 6:**
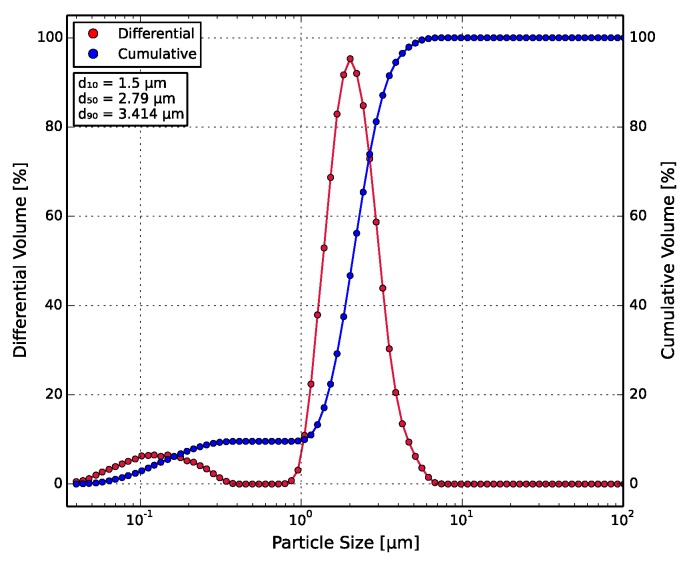
Particle size distribution of the BT particles, with red the differential and blue the cumulative volume. The particle distribution is bimodal, with the $d_{50}$ value lying at 2.79 μm.

The measurement of the particle size distribution of the barium titanate (BT) micro-powder was carried out in the Beckman Coulter LS-230 particle counter (Beckman Coulter, Brea, CA, USA). The particles were dispersed in isopropanol and ultrasonically treated to remove agglomerates. The particles exhibited a bimodal distribution, as shown in Figure 6, with a small fraction of the particles lying in the sub-micron range. The $d_{50}$ value was found to be 2.79 μm.

The bimodal distribution of the BT powder has implications on its dielectric performance, as there is evidence of the particle size influencing the permittivity of the ceramic [56,57].

### 4.2. Feedstock Preparation

All feedstock variants were kneaded at 180 ${}^{\circ }$C except for the 35 vol %, which was kneaded at at 210 ${}^{\circ }$C. The torque measurements at the end of the kneading process are shown in Table 1. Pure ABS was also kneaded as a reference.

### 4.3. Rheology

Rheological analysis was carried out in the Goettfert Rheograph 25 capillary rheometer (Goettfert GmbH, Buchen, Germany) to investigate the flow properties of the composites. Feedstock with up to 50 vol % were analyzed between shear-rates of 0.5 and 5000 s${}^{-1}$ at 180 ${}^{\circ }$C.

**Figure 7:**
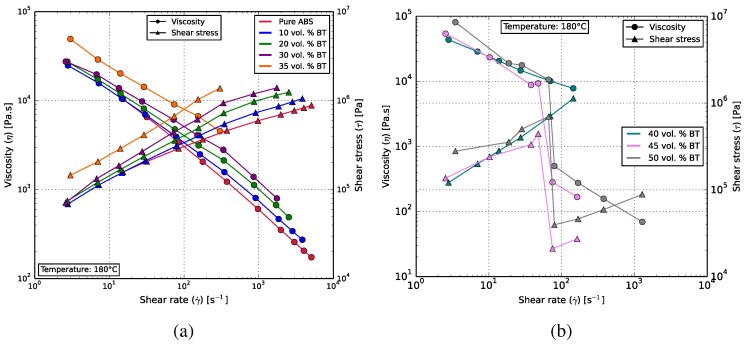
(**a**) Results obtained from the rheological analysis of the ABS-BT composites. The viscosity can be seen to decrease with increasing shear rate, indicating shear thinning behavior. The viscosity at any given shear rate is higher for samples with higher BT content. (**b**) The rheological behavior of composites with BT loadings of 40, 45 and 50 vol %. These could not be 3D printed. A stick-slip-like behavior can be seen for the 45 and 50 vol % samples.

Figure 7a shows the rheological behavior of the composites that could be 3D printed. Pure ABS, as well as the composites exhibit a non-Newtonian shear-thinning characteristic, with the BT content impeding the rate at which the dynamic viscosity decreases with increasing shear rates.

The composite with 40 vol % could not be printed reliably due to its brittleness, causing frequent filament breakages in the print-head. At filler ratios of 45 and 50 vol % (Figure 7b), the composites exhibited a sharp decrease in viscosity akin to the stick-slip phenomenon. The applied shear-rates are comparable to those experienced by the composite during filament extrusion, suggesting difficulties in filament extrusion. This was later confirmed, as the composites with these filler ratios were found to be challenging to extrude due to large voids and dimensional inaccuracies in the filament diameters.

### 4.4. Thermogravimetry

Thermogravimetric tests were performed in the Netzsch STA-409 differential calorimeter (Netzsch Group, Selb, Germany) to determine the effective ceramic content by mass for each composite. Pure and composite samples taken from feedstock and, where applicable, from 3D-printed specimens were heated from room temperature at 10 K/min–1000 ${}^{\circ }$C in an ambient atmosphere and held for 30 min.

**Figure 8:**
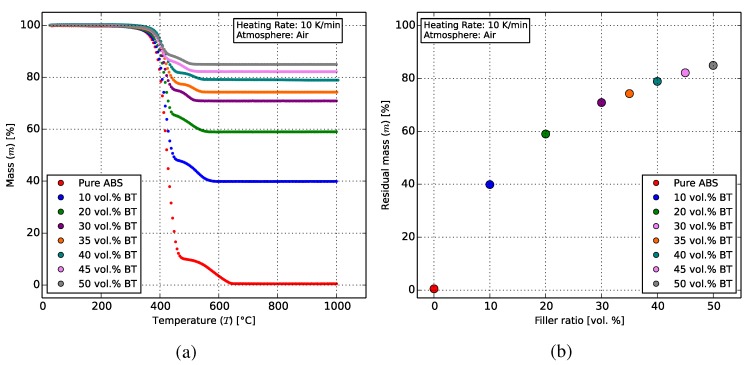
Thermogravimetric behavior of the ABS-BT composite feedstock with up to 50 vol % BT. (**a**) ABS can be seen to burn away between 400 and 650 ${}^{\circ }$C. The remaining ceramic masses remained consistent over the different phases of the process, with the 50 vol % composite correlating to 84.9 wt %. (**b**) The remaining ceramic masses after thermogravimetry.

Figure 8a shows that for all materials, ABS decomposes and burns away between 400 and 650 ${}^{\circ }$C, which is expected due to its organic nature. The remaining masses were then correlated with the calculated volumetric contents of the samples.

The remaining ceramic masses for all composites (Figure 8b) correlate well with theory, since the density of BT (6.02 g/cm${}^{3}$) is around six-times that of ABS (1.04 g/cm${}^{3}$). The theoretical and measured values are indicated in Table 2 and lie within the error margins of the measurement device.

Samples from extruded filaments and 3D prints with up to 35 vol % BT were thermogravimetrically analyzed and produced similar results, indicating negligible thermal influence, e.g., polymer decomposition at high temperature during printing, on the polymer-ceramic ratio throughout the process.

## 5. Structural Characterization

### 5.1. Print Parameter Optimization

Print parameters were optimized for each of the composites for a layer thicknesses of 0.2 mm. Several print parameters were kept constant for all specimens based on [58] and are listed in Table 3.

The influence of part orientation and nozzle temperatures was investigated and optimized for pure ABS test structures. These were then used as standard parameters for the composites.

#### 5.1.1. Part Orientation

Pure ABS test samples were printed in laid-down and upright orientations to investigate the influence of the part orientation on the mechanical strength of the sample. These longitudinal or transverse orientations caused the applied tensile force to be, respectively, parallel or perpendicular to the print layers of the specimen (Figure 4b). Preliminary tests showed that intermediate print orientations, such as 30${}^{\circ }$ and 45${}^{\circ }$, were found to be more challenging to print without supports due to their overhanging shape.

**Figure 9:**
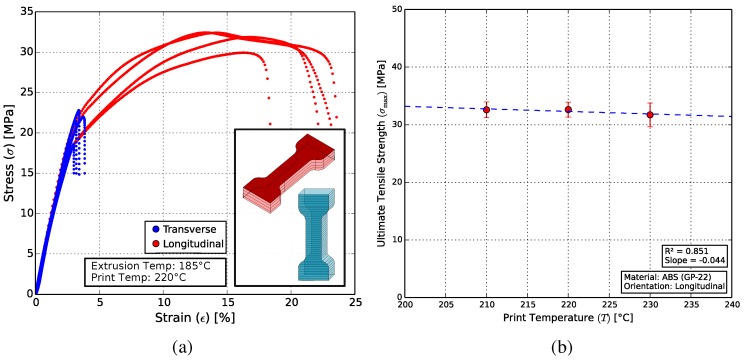
(**a**) The influence of print orientation on the mechanical strength of a tensile test specimen. Longitudinally-oriented samples (red) showed a relatively large region of plastic deformation. Transversely-printed structures (blue), on the other hand, failed at much lower forces without a plastic deformation region. Inset: Illustrations of the print layers for the two print orientations. (**b**) The influence of print temperature on the ultimate tensile strength (UTS) of the tensile specimens is negligible.

Figure 9a shows that the longitudinally-printed test structures exhibit a relatively large region of plastic deformation. Transversely-printed samples showed a comparatively brittle characteristic. This is due to dislocations at the print layer boundaries, which lie perpendicular to the direction of the applied force. Owing to their plasticity, longitudinally-oriented specimens were used as the standard for further experiments.

#### 5.1.2. Print Temperature

The influence of the printer’s nozzle temperature on the tensile strength of pure ABS samples was tested. Figure 9b shows that this influence is negligible in the temperature range between the manufacturer-recommended range of 210 and 230 ${}^{\circ }$C. The composite test specimens were printed at 230 ${}^{\circ }$C, to accommodate for the increased heat capacity expected due to the presence of the ceramic particles.

### 5.2. Microscopy

Microscopy was performed using the Zeiss Axioplan 2 microscope (Carl Zeiss Microscopy GmBH, Jena, Germany). 3D-printed tensile specimen cross-sections with up to 35 vol % BT were imaged at 20× and 50× magnification.

**Figure 10:**
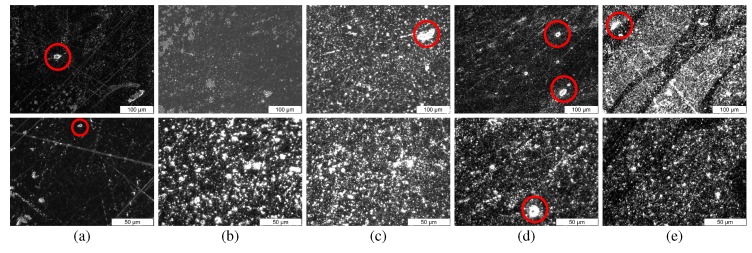
Microscope images of tensile test specimen cross-sections of (**a**) pure ABS, (**b**) 10 vol %, (**c**) 20 vol %, (**d**) 30 vol % and (**e**) 35 vol % at (top row) 20× and (bottom row) 50× zoom. Some visible voids have been marked with red circles.

The images in Figure 10 have been contrast enhanced and show the presence of voids on the specimen cross-section, shown in red. The BT particles can also be seen to agglomerate in certain regions.

### 5.3. Tensile Characterization

Tensile test specimens, as described in Section 3.1.1, were analyzed for the influence of the ceramic content on the ultimate tensile strength (UTS) and compared with those for pure ABS. The specimens were printed at 230 ${}^{\circ }$C and were longitudinally oriented.

**Figure 11:**
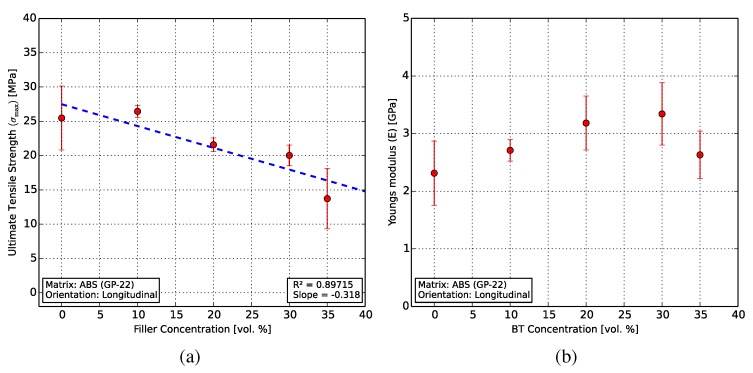
Results from the tensile characterization. (**a**) A linearly decreasing UTS ($\sigma_{max}$) with increasing BT filler concentration was observed from 25.5 to 13.7 MPa. (**b**) The Young’s modulus of the composites showed a slight increase compared to that of ABS.

Figure 11a shows the UTS linearly decreasing from 25.5 MPa for pure ABS to 13.7 MPa for the composite with 35 vol % BT. This decrease can be attributed to the presence of an increasing number of ceramic particles which hinder the polymer matrix, reducing bulk ductility. The particles can also form clumps and agglomerates, and regions with high ceramic content lead to increased brittleness. Additionally, the presence of air pockets and voids in the specimen cross-sections (see Figure 10) also reduces the structural integrity of the specimens. These voids can be carried over into the specimen from those produced during filament extrusion and can also appear on inter-layer boundaries during 3D printing. Longer kneading times and de-agglomeration of the ceramic particles, as well as the use of smaller filler particles can improve these results.

The UTS of pure ABS was found to be at around 40% of the manufacturer-provided values (65 MPa for Terluran^®^GP-22). When compared to 3D-printed ABS specimens from the literature using similar print parameters [30,58,59], the results are comparable (20, 32.6 and 16 MPa, respectively).

Figure 11b shows the influence of the filler on the secant Young’s modulus, measured between the start and the end of the linear deformation region. The Young’s modulus of ABS averages 2.3 GPa and is comparable to those of the composites, with their mean values lying between 2.6 and 3.3 GPa.

### 5.4. Flexural Characterization

**Figure 12:**
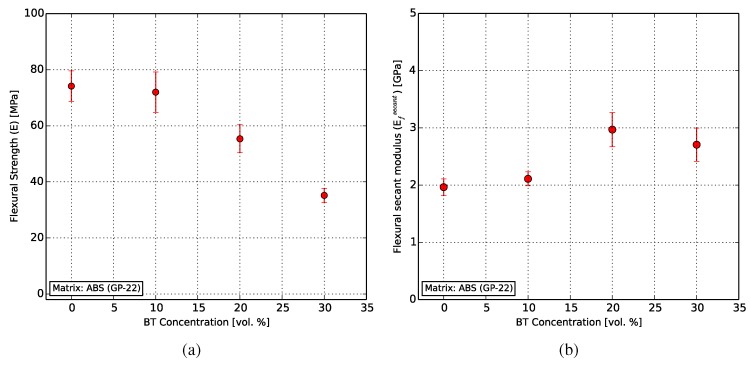
Flexural behavior of the composites, with (**a**) increasing filler content decreasing the flexural strength of the composite and (**b**) increasing the flexural secant modulus of elasticity.

Flexural characterization was carried out on the 3D-printed test specimens (see Section 3.1.2) to investigate the influence of the filler on the flexural strength, using Equation (1). The increasing filler ratio shows a decrease in the flexural strength from 74.14 MPa for pure ABS to 35.18 MPa for the specimens with 30 vol % BT, as shown in Figure 12a.

This decrease was expected due to the influence of the ceramic particles decreasing the flexibility of the polymer matrix along the printed layers. The effect of the non-homogeneous filler particle distribution can be seen here, as well, as the pure ABS and 10 vol % BT points lie close to each other.

Figure 12b shows the behavior of the flexural secant modulus of elasticity for the composites, calculated using Equation (2). It can be seen to increase from 1.97 GPa for pure ABS (compared to 1.65 GPa from [59]) to a maximum of 2.97 GPa for the 20 vol % composite.

## 6. Functional Characterization

**Figure 13:**
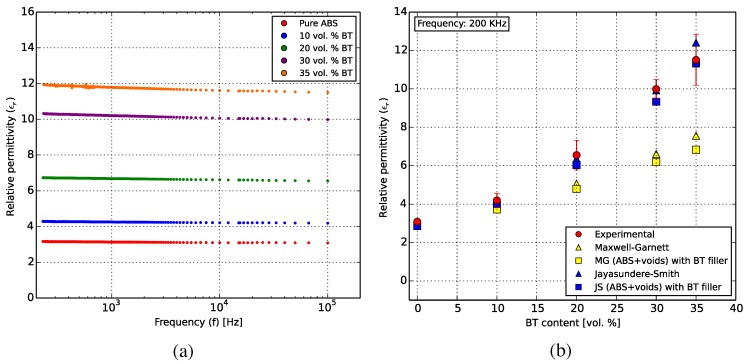
(**a**) The dielectric behavior of pure ABS and the ABS-BT composites over a frequency sweep from around 250 Hz–200 kHz. (**b**) A correlation of the obtained results (red circles) with the Maxwell-Garnett (yellow) and Jayasundere-Smith (blue) effective medium approximations at 200 kHz, with and without the effect of voids as a part of the matrix material in the samples (triangles and squares, respectively).

Dielectric characterization was performed for the test specimens (as described in Section 3.2) over a frequency range between 250 Hz and 200 kHz at room temperature. Figure 13 shows the results from the dielectric analysis. The relative permittivity $\epsilon_{r}$ was calculated using Equation (3) and correlated with the Maxwell-Garnett (Equation (4)) and Jayasundere-Smith (Equation (5)) effective medium approximations at 200 kHz.

The relative permittivity of the composites was seen to increase with increasing BT filler ratio while decreasing logarithmically with frequency, as shown in Figure 13a. At 200 kHz, the ABS-35 vol % BT specimens showed a mean $\epsilon_{r}$ of 11.5. These values are equal to or better than those of a recent study [11] of the behavior of 3D-printed ABS-BT composites in the GHz regime. A further increase in the filler content and measurements at higher temperatures will predictably exhibit higher permittivities.

Figure 13b shows the correlation of the measured permittivities with the Maxwell-Garnett (MG) and Jayasundere-Smith (JS) effective medium models based on the permittivities of ABS and BT from the literature, as discussed in Section 3.2. Both models and the measured data predict the expected non-linear increase in permittivity at higher filler ratios. The discrepancies between the modeled and measured values could be due to the particle size dependency of permittivity and the bimodal particle size distribution of BT (Figure 6), as sub-micron particles have previously been shown to exhibit higher permittivities. Moreover, neither model takes the applied frequency into account, which also affects the measured permittivity. At higher frequencies, the measured data would move closer to the MG model.

The influence of the air pocket volume on the permittivity was analyzed by back-calculating the volume fraction of air in each of the samples (see Equation (6)). These are shown in Table 4.

The pure ABS sample showed an air volume fraction of 2.1 vol %. For the composites, it was seen to increase with BT content, with the maximum at 11.9 vol % for the 35 vol % sample.

The MG and JS models were additionally used to correlate the effect of the voids on the permittivity by taking the ABS-air combination as the matrix, filled with BT particles. (Figure 13b squares). Both models agree well with the measured data at low filler contents. The void-influenced permittivity reduction for the 35 vol % composite is 9% in the JS model and 10% in the MS approximation. The MG model’s predictions fall off at higher filler ratios, while the JS model showed continued agreement and could be used for predicting permittivities of composites with higher filler content for this material system. The MG model could be more accurate at higher frequencies and for different BT grain sizes.

## 7. Conclusions

In this work, a comprehensive four-phase process-chain for the development and characterization of a 3D printable functional composite was described. The process showed repeatability through the various development and characterization steps. It was shown to be compatible with a commercially available FDM printer and can be adopted for other polymer-based composites, as well as other additive rapid prototyping technologies.

The ABS-BT composites investigated in this study were prepared in filler ratios of up to 50 vol % and tested and were found to be reliably printable up to 35 vol %. Due to their brittleness, filaments with 40 vol % could not be printed. At 45 and 50 vol %, the composites showed stick-slip behavior and were not repeatably extrudable.

Structural analysis of the 3D-printed composites revealed an expected decrease in tensile and flexural strength with increasing ceramic content. The Young’s moduli of the composites increased by up to 69% compared to those of the pure polymer specimens, whereas the flexural secant moduli showed an increase of up to 66%. Dielectric characterization showed a non-linearly increasing relative permittivity with filler concentration up to 11.5 at 200 kHz and was shown to correlate well with the Jayasundere-Smith effective medium approximation, including the effect of the voids in the samples.

Inhomogeneous filler particle distribution is an important factor in the premature mechanical failure of the printed specimens and can be improved by longer kneading times. The presence of voids and air pockets is an inherent result of FDM’s layer-by-layer printing method, and while not completely avoidable, it can be reduced by drying the feedstock before filament extrusion.

3D-printing of ABS-BT composites with higher filler ratios would require the use of viscosity-reducing additives that would allow the composite to retain a parabolic flow profile during filament extrusion, leading to dimensionally accurate filaments. The use of rubbery materials as conveying wheels in the print-head could also be useful in avoiding filament breakage in the 3D-printer. If these challenges can be overcome, the expected non-linear increase in permittivity with filler content can make the ABS-BT composites potentially useful as 3D-printed passive antennas.

## Figures and Tables

**Table 1:** Torque values for the composite feedstock at the end of the kneading step (* @210 °C).

BT Ratio (vol %)	End Torque (N·m)
0	24.0
10	26.0
20	27.1
30	28.6
35 *	15.7 *
40	32.4
45	34.9
50	37.7

**Table 2:** Mass ratios obtained after thermogravimetry compared with theoretical values.

BT Ratio (vol %)	Theoretical (wt %)	Measured (wt %)	Back-Calculated (vol %)
0	0	0.47
10	38.4	39.9	10.5
20	58.5	59.0	20.3
30	70.8	70.8	30.2
35	75.2	74.2	33.9
40	79.0	79.0	40.0
45	82.2	82.1	44.9
50	85.0	84.9	50.0

**Table 3:** 3D print parameters kept constant throughout the design of experiments.

Number of Shells	Nozzle Diameter (μm)	Raster Angle (°)	Raster Width (μm)	Infill (%)	Build-Table Temperature (°C)
3	400	45	200	100	110

**Table 4:** Void volumes inside the dielectric samples, with the percentage reduction in permittivity due to the voids as predicted by the MG and JS models.

BT Ratio (vol %)	Voids (vol %)	MG Reduction (%)	JS Reduction (%)
0	2.1	1.7	1.7
10	4.4	3.8	3.6
20	6.5	5.3	5.2
30	7.8	6.6	6.2
35	11.9	10.0	9.0

## Abbreviations

The following abbreviations are used in this manuscript:

ARP	Additive rapid prototyping
RP	Rapid prototyping
SL	Stereolithography
SLS	Selective laser sintering
PJM	Polyjet modeling
FDM	Fused deposition modeling
FFF	Fused filament fabrication
ABS	Acrylonitrile butadiene styrene
BT	Barium titanate
UTS	Ultimate tensile strength
MG	Maxwell-Garnett
JS	Jayasundere-Smith
